# *N*-(2-Bromo­benz­yl)cinchoninium bromide

**DOI:** 10.1107/S160053681203646X

**Published:** 2012-08-31

**Authors:** Agnieszka Skórska-Stania, Magdalena Jezierska-Zięba, Barbara Kąkol, Michał Fedoryński, Barbara J. Oleksyn

**Affiliations:** a Faculty of Chemistry, Jagiellonian University, Ingardena 3, 30-060 Kraków, Poland; b Industrial Chemistry Research Institute, Rydygiera 8, 01-793 Warsaw, Poland; c Faculty of Chemistry, Warsaw University of Technology, Noakowskiego 3, 00-664 Warsaw, Poland

## Abstract

The title compound {systematic name: 1-(2-bromo­benz­yl)-5-ethenyl-2-[hy­droxy(quinolin-4-yl)meth­yl]-1-aza­bicyclo­[2.2.2]octan-1-ium bromide}, C_26_H_28_BrN_2_O^+^·Br^−^, is a chiral quater­nary ammonium salt of one of the *Cinchona* alkaloids. The planes of the quinoline and of the bromo­benzyl substituent are inclined to one another by 9.11 (9)°. A weak intra­molecular C—H⋯O hydrogen bond occurs. The crystal structure features strong O—H⋯Br hydrogen bonds and weak C—H⋯Br inter­actions.

## Related literature

For the structure of cinchonine base and its derivatives, see: Oleksyn *et al.* (1979 ▶); Dolling *et al.* (1984 ▶). For crystal structures of other selected *Cinchona* alkaloid derivatives with bulky substituents at the quinuclidine nitro­gen atom, see: Song *et al.* (2005 ▶); Kawai *et al.* (2009 ▶); Jew *et al.* (2002 ▶); Matoba *et al.* (2010 ▶). For the effect of the substituent on the activity of the title catalyst, see: Jezierska-Zięba *et al.* (2010 ▶). 
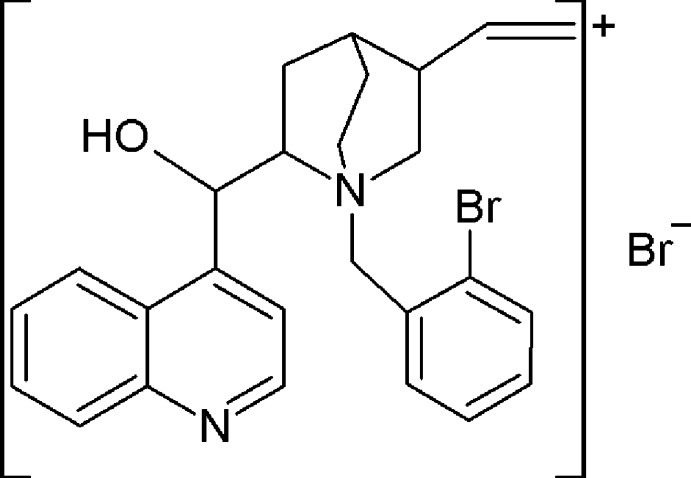


## Experimental

### 

#### Crystal data

C_26_H_28_BrN_2_O^+^·Br^−^
*M* *_r_* = 544.30
Orthorhombic, 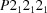

*a* = 7.2313 (1) Å
*b* = 16.2545 (1) Å
*c* = 20.2466 (2) Å
*V* = 2379.81 (4) Å^3^
*Z* = 4
Mo *K*α radiation
μ = 3.43 mm^−1^
*T* = 295 K
0.2 × 0.15 × 0.1 mm

#### Data collection

Nonius KappaCCD diffractometer
Absorption correction: multi-scan (*DENZO* and *SCALEPACK*; Otwinowski & Minor 1997 ▶) *T*_min_ = 0.547, *T*_max_ = 0.726
64183 measured reflections
5437 independent reflections
4879 reflections with *I* > 2σ(*I*)
*R*_int_ = 0.039

#### Refinement

*R*[*F* ^2^ > 2σ(*F* ^2^)] = 0.034
*wR*(*F* ^2^) = 0.071
*S* = 1.06
5437 reflections
283 parameters
H atoms treated by a mixture of independent and constrained refinement
Δρ_max_ = 0.40 e Å^−3^
Δρ_min_ = −0.42 e Å^−3^
Absolute structure: Flack (1983 ▶), 2320 Friedel pairs
Flack parameter: 0.020 (8)

### 

Data collection: *COLLECT* (Nonius, 1998 ▶); cell refinement: *SCALEPACK*; data reduction: *DENZO* (Otwinowski & Minor, 1997 ▶) and *SCALEPACK* (Otwinowski & Minor, 1997 ▶); program(s) used to solve structure: *SHELXS97* (Sheldrick, 2008 ▶); program(s) used to refine structure: *SHELXL97* (Sheldrick, 2008 ▶); molecular graphics: *ORTEP-3* (Farrugia, 1997 ▶); software used to prepare material for publication: *WinGX* (Farrugia, 1999 ▶).

## Supplementary Material

Crystal structure: contains datablock(s) global, I. DOI: 10.1107/S160053681203646X/rk2374sup1.cif

Structure factors: contains datablock(s) I. DOI: 10.1107/S160053681203646X/rk2374Isup2.hkl

Supplementary material file. DOI: 10.1107/S160053681203646X/rk2374Isup3.mol

Supplementary material file. DOI: 10.1107/S160053681203646X/rk2374Isup4.cml

Additional supplementary materials:  crystallographic information; 3D view; checkCIF report

**Fig. 3.**
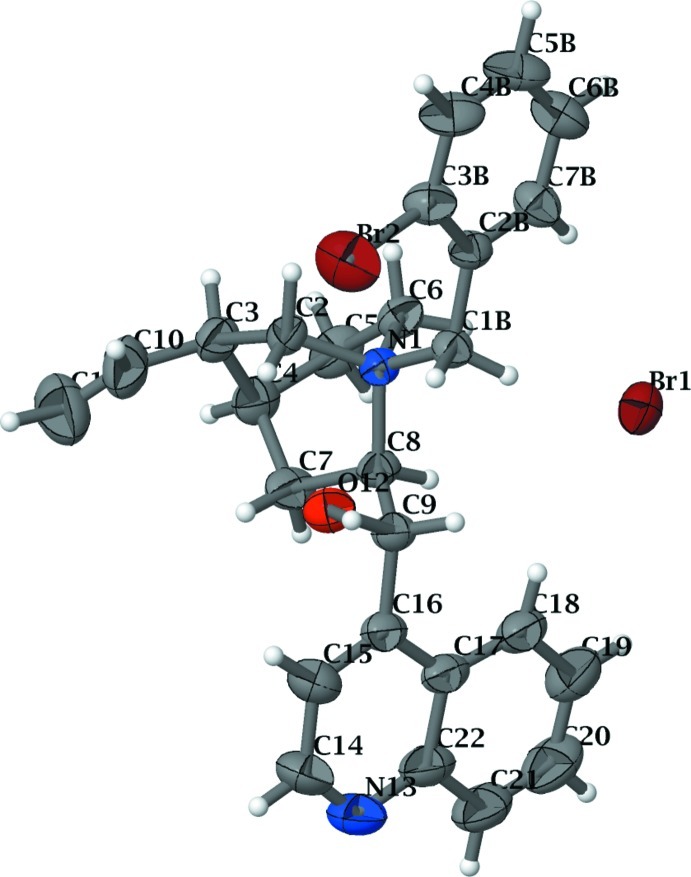
Enhanced figure.

Enhanced figure: interactive version of Fig. 3

## Figures and Tables

**Table 1:** Hydrogen-bond geometry (Å, °)

*D*—H⋯*A*	*D*—H	H⋯*A*	*D*⋯*A*	*D*—H⋯*A*
O12—H12⋯Br1^i^	0.81 (4)	2.38 (4)	3.179 (2)	173 (3)
C2—H2*B*⋯O12	0.97	2.32	2.997 (4)	126
C6—H6*A*⋯Br1	0.97	2.88	3.797 (3)	159
C18—H18⋯Br1	0.93	2.96	3.758 (4)	145

Symmetry code: (i) 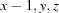.

**Fig. 1.**
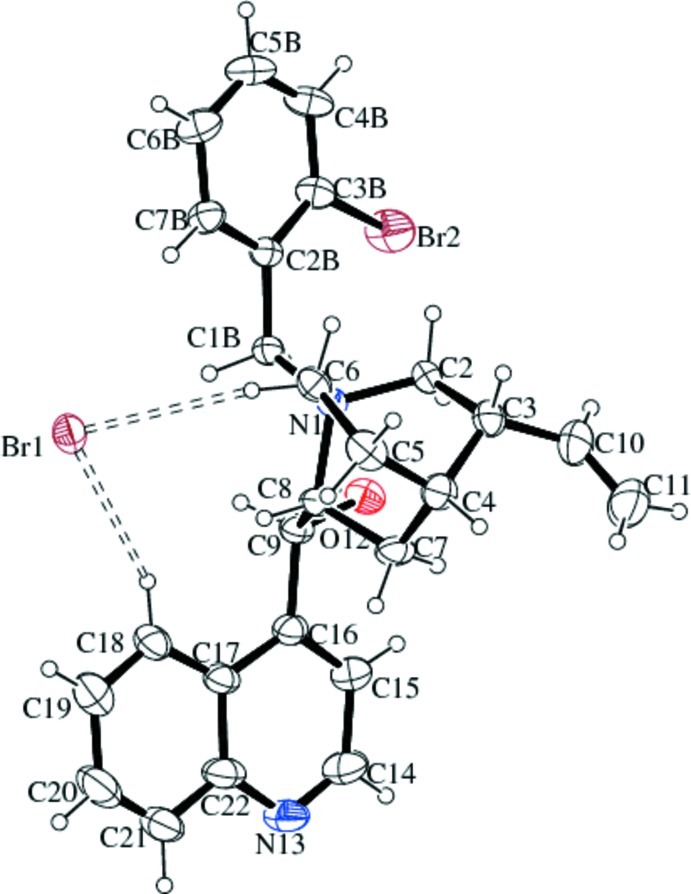
The asymmetric unit of the title compound with the atom numbering scheme. Displacement ellipsoids are drawn at the 30% probability level. H atoms are shown as spheres of arbitrary radii. The C—H···Br hydrogen bonds are in dashed lines.

**Fig. 2.**
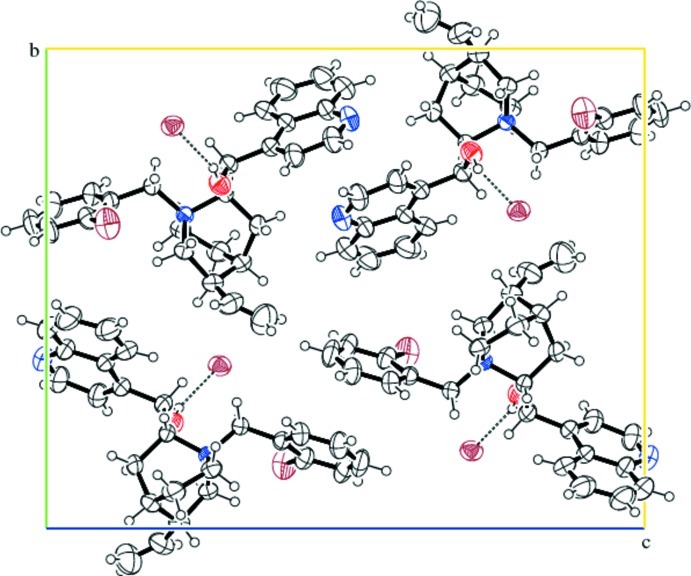
The packing viewed along a axis with strong hydrogen bonds shown by dashed lines.

## supplementary crystallographic information

### Comment

The *Cinchona* alkaloids with bulky substituents at quinuclidine nitrogen atom (N1), as the potential catalysts, have been studied crystallographically in the last three decades: Dolling *et al.* (1984); Song *et al.* (2005); Kawai *et al.* (2009); Jew *et al.*(2002); Matoba *et al.* (2010). The asymmetric unit of the title compound is composed of *N*–(2–bromobenzyl)cinchoninium cation and bromide anion (Fig. 1). The title cinchonine derivative was used as a catalyst in the asymmetric Darzens condensation between benzaldehyde and alkylchloroacetates: Jezierska-Zięba *et al.* (2010).

The conformational features of the title compound are similar to those of the related parent structure of cinchonine base (Oleksyn *et al.*, 1979), with exception of the orientation of the vinyl group towards the quinuclidine moiety. The packing is dominated by the strong hydrogen bonding O12—H···Br1 (Fig. 2). The pairs cation–anion interact with each other *via* short contacts C—H···Br1, forming chains parallel to [1 0 0]. The chains are strengthened by short C—H···Br2 contacts. The oxygen atom (O12), is an acceptor in weak intramolecular hydrogen bonds. The hydrogen bond geometry is given in Table 1.

The disorder of the vinyl groups occurs in almost every molecular structure of *Cinchona* alkaloids, we have determined. The vinyl group (*i.e.* C10 and C11 atoms) is present on the periphery of the whole molecule, so it has ability to move. The conformation of the vinyl moiety, which we present here, is close to the potential energy minimum and is frequently observed in the structures of *erythro Cinchona* alkaloids.

### Experimental

A mixture of cinchonine (2.95 g, 0.01 mol) and 2–bromobenzylbromide (2.5 g, 0.01 mol) in toluene (40 ml) was stirred and heated at 353 K for 4 h. After cooling to room temperature, hexane (100 ml) was added and the mixture was stirred for 10 h. The precipitated crystals were collected by suction filtration, washed with acetonitrile and dried to give *N*–(2–bromobenzyl)cinchoninium bromide (5.25 g, 97%, m.p. 430 K). Single crystals suitable for X–ray diffraction study were obtained from ethanol by slow evaporation at room temperature.

### Refinement

All hydrogen atoms were found on a difference Fourier maps and refined using a riding model with C—H = 0.93Å and *U*_iso_(H) = 1.2*U*_eq_(C) for aromatic hydrogen atoms, C—H = 0.97Å and *U*_iso_(H) = 1.2*U*_eq_(C) for methylene groups and C—H = 0.98Å and *U*_iso_(H) = 1.2*U*_eq_(C) for methine groups. The O based atom H12 was refined with *U*_iso_(H) = 1.2*U*_eq_(O).

### Crystal data

**Table:**

C_26_H_28_BrN_2_O^+^·Br^−^	*D*_x_ = 1.519 Mg m^−^^3^
*M**_r_* = 544.30	Melting point: 430 K
Orthorhombic, *P*2_1_2_1_2_1_	Mo *K*α radiation, λ = 0.71073 Å
Hall symbol: P 2ac 2ab	Cell parameters from 3135 reflections
*a* = 7.2313 (1) Å	θ = 1.0–27.5°
*b* = 16.2545 (1) Å	µ = 3.43 mm^−^^1^
*c* = 20.2466 (2) Å	*T* = 295 K
*V* = 2379.81 (4) Å^3^	Prism, colourless
*Z* = 4	0.2 × 0.15 × 0.1 mm
*F*(000) = 1104	

### Data collection

**Table:**

Nonius KappaCCD diffractometer	5437 independent reflections
Radiation source: fine–focus sealed tube	4879 reflections with *I* > 2σ(*I*)
Graphite monochromator	*R*_int_ = 0.039
Detector resolution: 9 pixels mm^-1^	θ_max_ = 27.5°, θ_min_ = 2.7°
CCD rotation images, thick slices scans	*h* = −9→9
Absorption correction: multi-scan (*DENZO* and *SCALEPACK*; Otwinowski & Minor 1997)	*k* = −20→21
*T*_min_ = 0.547, *T*_max_ = 0.726	*l* = −26→26
64183 measured reflections	

### Refinement

**Table:**

Refinement on *F*^2^	Secondary atom site location: difference Fourier map
Least-squares matrix: full	Hydrogen site location: difference Fourier map
*R*[*F*^2^ > 2σ(*F*^2^)] = 0.034	H atoms treated by a mixture of independent and constrained refinement
*wR*(*F*^2^) = 0.071	*w* = 1/[σ^2^(*F*_o_^2^) + (0.023*P*)^2^ + 1.5842*P*] where *P* = (*F*_o_^2^ + 2*F*_c_^2^)/3
*S* = 1.06	(Δ/σ)_max_ = 0.001
5437 reflections	Δρ_max_ = 0.40 e Å^−^^3^
283 parameters	Δρ_min_ = −0.42 e Å^−^^3^
0 restraints	Absolute structure: Flack (1983), 2320 Friedel pairs
Primary atom site location: structure-invariant direct methods	Flack parameter: 0.020 (8)

### Special details

**Table:**

Geometry. All s.u.'s (except the s.u. in the dihedral angle between two l.s. planes) are estimated using the full covariance matrix. The cell s.u.'s are taken into account individually in the estimation of s.u.'s in distances, angles and torsion angles; correlations between s.u.'s in cell parameters are only used when they are defined by crystal symmetry. An approximate (isotropic) treatment of cell s.u.'s is used for estimating s.u.'s involving l.s. planes.

### Fractional atomic coordinates and isotropic or equivalent isotropic displacement parameters (Å^2^)

**Table:**

	*x*	*y*	*z*	*U*_iso_*/*U*_eq_	
C1B	0.1042 (4)	0.78679 (16)	0.82411 (13)	0.0396 (6)	
H1BA	−0.0247	0.7713	0.8194	0.048*	
H1BB	0.1776	0.7374	0.8182	0.048*	
C2B	0.1337 (4)	0.81793 (16)	0.89340 (13)	0.0393 (6)	
C3B	−0.0039 (4)	0.85398 (19)	0.93115 (14)	0.0508 (7)	
C4B	0.0243 (5)	0.8774 (2)	0.99629 (16)	0.0647 (9)	
H4BA	−0.0705	0.9017	1.0204	0.078*	
C5B	0.1937 (5)	0.8641 (3)	1.02470 (16)	0.0701 (11)	
H5BA	0.2148	0.8804	1.0681	0.084*	
C6B	0.3319 (5)	0.8269 (2)	0.98952 (16)	0.0643 (9)	
H6BA	0.4461	0.8174	1.0092	0.077*	
C7B	0.3019 (4)	0.8036 (2)	0.92451 (15)	0.0497 (7)	
H7BA	0.3964	0.7777	0.9012	0.06*	
C2	0.0284 (4)	0.92222 (15)	0.77020 (13)	0.0426 (6)	
H2A	0.0258	0.9448	0.8146	0.051*	
H2B	−0.0967	0.9064	0.7584	0.051*	
C3	0.0976 (5)	0.98821 (17)	0.72183 (15)	0.0514 (8)	
H3	0.167	1.0292	0.7472	0.062*	
C4	0.2313 (5)	0.94693 (18)	0.67445 (15)	0.0528 (7)	
H4	0.2626	0.9844	0.6382	0.063*	
C5	0.4058 (4)	0.92317 (19)	0.71277 (18)	0.0577 (8)	
H5A	0.4749	0.9723	0.7243	0.069*	
H5B	0.484	0.8886	0.6855	0.069*	
C6	0.3508 (4)	0.87670 (18)	0.77580 (14)	0.0453 (7)	
H6A	0.4327	0.8302	0.7824	0.054*	
H6B	0.3613	0.9128	0.8138	0.054*	
C7	0.1435 (5)	0.86819 (19)	0.64724 (13)	0.0534 (8)	
H7A	0.2132	0.8489	0.6093	0.064*	
H7B	0.0178	0.8793	0.6331	0.064*	
C8	0.1433 (4)	0.80217 (16)	0.70143 (13)	0.0395 (6)	
H8	0.2581	0.7706	0.6967	0.047*	
C9	−0.0178 (4)	0.74086 (17)	0.69529 (13)	0.0427 (6)	
H9	0.0001	0.696	0.727	0.051*	
C10	−0.0719 (7)	1.0313 (2)	0.6916 (2)	0.0775 (11)	
H10	−0.1767	1.033	0.7182	0.093*	
C11	−0.0879 (8)	1.0640 (3)	0.6368 (2)	0.1018 (16)	
H11A	0.0116	1.0645	0.6077	0.122*	
H11B	−0.1995	1.0881	0.6247	0.122*	
C14	−0.1798 (7)	0.6905 (3)	0.52105 (18)	0.0800 (13)	
H14	−0.2847	0.7015	0.496	0.096*	
C15	−0.1729 (6)	0.7227 (2)	0.58609 (17)	0.0644 (9)	
H15	−0.2684	0.7557	0.6018	0.077*	
C16	−0.0262 (5)	0.70516 (17)	0.62510 (14)	0.0487 (7)	
C17	0.1183 (5)	0.65446 (18)	0.59904 (14)	0.0508 (7)	
C18	0.2769 (5)	0.62946 (19)	0.63382 (17)	0.0600 (9)	
H18	0.2882	0.643	0.6783	0.072*	
C19	0.4150 (6)	0.5857 (2)	0.6038 (2)	0.0775 (11)	
H19	0.5204	0.5712	0.6274	0.093*	
C20	0.3970 (7)	0.5627 (3)	0.5368 (2)	0.0876 (14)	
H20	0.4914	0.5335	0.5163	0.105*	
C21	0.2452 (8)	0.5826 (2)	0.50268 (19)	0.0803 (12)	
H21	0.2351	0.5664	0.4588	0.096*	
C22	0.1005 (6)	0.6277 (2)	0.53171 (16)	0.0637 (10)	
Br2	−0.24868 (5)	0.86541 (3)	0.898389 (19)	0.07787 (13)	
N1	0.1527 (3)	0.84692 (14)	0.76881 (10)	0.0357 (5)	
N13	−0.0494 (6)	0.6463 (2)	0.49364 (14)	0.0778 (9)	
O12	−0.1842 (3)	0.78260 (13)	0.71029 (12)	0.0520 (5)	
H12	−0.255 (6)	0.750 (2)	0.7269 (17)	0.062*	
Br1	0.55118 (5)	0.661427 (19)	0.789365 (17)	0.05560 (9)	

### Atomic displacement parameters (Å^2^)

**Table:**

	*U*^11^	*U*^22^	*U*^33^	*U*^12^	*U*^13^	*U*^23^
C1B	0.0480 (16)	0.0380 (14)	0.0328 (13)	0.0005 (11)	0.0036 (11)	0.0000 (11)
C2B	0.0437 (14)	0.0398 (15)	0.0344 (13)	−0.0034 (11)	0.0035 (12)	−0.0023 (11)
C3B	0.0480 (16)	0.0590 (19)	0.0454 (15)	−0.0050 (13)	0.0062 (12)	−0.0073 (13)
C4B	0.068 (2)	0.082 (2)	0.0445 (16)	−0.0092 (19)	0.0197 (16)	−0.0180 (16)
C5B	0.083 (3)	0.090 (3)	0.0379 (16)	−0.018 (2)	0.0009 (16)	−0.0157 (17)
C6B	0.059 (2)	0.086 (3)	0.0477 (17)	−0.008 (2)	−0.0115 (16)	−0.0009 (18)
C7B	0.0453 (17)	0.0621 (18)	0.0418 (15)	0.0010 (14)	0.0006 (12)	0.0023 (14)
C2	0.0485 (15)	0.0354 (13)	0.0441 (15)	0.0065 (12)	0.0015 (12)	−0.0036 (10)
C3	0.065 (2)	0.0362 (13)	0.0526 (18)	−0.0020 (13)	0.0017 (15)	0.0007 (12)
C4	0.069 (2)	0.0422 (15)	0.0477 (16)	−0.0098 (16)	0.0070 (16)	0.0069 (13)
C5	0.0567 (19)	0.0487 (16)	0.068 (2)	−0.0120 (13)	0.0198 (17)	−0.0014 (16)
C6	0.0425 (14)	0.0407 (14)	0.0527 (17)	−0.0032 (12)	0.0038 (13)	−0.0053 (13)
C7	0.080 (2)	0.0486 (16)	0.0316 (13)	−0.0104 (17)	0.0094 (14)	0.0025 (12)
C8	0.0498 (15)	0.0366 (13)	0.0320 (13)	−0.0022 (11)	0.0054 (12)	−0.0038 (11)
C9	0.0507 (16)	0.0405 (13)	0.0369 (14)	−0.0038 (12)	−0.0004 (12)	−0.0043 (11)
C10	0.105 (3)	0.055 (2)	0.072 (2)	0.013 (2)	0.007 (2)	0.0189 (17)
C11	0.101 (4)	0.117 (4)	0.088 (3)	0.040 (3)	−0.004 (3)	0.014 (3)
C14	0.109 (3)	0.082 (3)	0.049 (2)	0.001 (2)	−0.027 (2)	−0.0106 (19)
C15	0.077 (2)	0.069 (2)	0.0479 (18)	0.0013 (19)	−0.0131 (17)	−0.0070 (16)
C16	0.067 (2)	0.0406 (14)	0.0389 (14)	−0.0069 (14)	−0.0031 (14)	−0.0022 (11)
C17	0.075 (2)	0.0375 (14)	0.0400 (14)	−0.0082 (14)	0.0042 (14)	−0.0044 (13)
C18	0.081 (3)	0.0424 (16)	0.0566 (18)	−0.0025 (18)	0.0016 (17)	−0.0104 (14)
C19	0.085 (3)	0.059 (2)	0.089 (3)	0.011 (2)	0.005 (2)	−0.014 (2)
C20	0.113 (4)	0.068 (2)	0.082 (3)	0.015 (3)	0.031 (3)	−0.017 (2)
C21	0.122 (4)	0.063 (2)	0.056 (2)	0.006 (3)	0.015 (3)	−0.0161 (18)
C22	0.100 (3)	0.0491 (17)	0.0424 (16)	−0.0123 (19)	0.0058 (17)	−0.0077 (14)
Br2	0.04306 (16)	0.1171 (3)	0.0734 (2)	0.0077 (2)	0.01098 (18)	−0.0085 (2)
N1	0.0380 (11)	0.0334 (11)	0.0357 (11)	−0.0020 (10)	0.0027 (9)	−0.0019 (9)
N13	0.115 (3)	0.073 (2)	0.0450 (14)	−0.002 (2)	−0.0107 (18)	−0.0150 (14)
O12	0.0477 (11)	0.0548 (12)	0.0536 (12)	−0.0049 (9)	0.0028 (11)	−0.0070 (11)
Br1	0.05621 (17)	0.04800 (15)	0.06259 (17)	0.00830 (15)	0.00511 (16)	0.00209 (15)

### Geometric parameters (Å, º)

**Table:**

C1B—C2B	1.507 (4)	C7—C8	1.535 (4)
C1B—N1	1.527 (3)	C7—H7A	0.97
C1B—H1BA	0.97	C7—H7B	0.97
C1B—H1BB	0.97	C8—C9	1.538 (4)
C2B—C3B	1.385 (4)	C8—N1	1.548 (3)
C2B—C7B	1.390 (4)	C8—H8	0.98
C3B—C4B	1.388 (4)	C9—O12	1.414 (4)
C3B—Br2	1.899 (3)	C9—C16	1.536 (4)
C4B—C5B	1.370 (5)	C9—H9	0.98
C4B—H4BA	0.93	C10—C11	1.235 (6)
C5B—C6B	1.368 (5)	C10—H10	0.93
C5B—H5BA	0.93	C11—H11A	0.93
C6B—C7B	1.387 (4)	C11—H11B	0.93
C6B—H6BA	0.93	C14—N13	1.309 (5)
C7B—H7BA	0.93	C14—C15	1.418 (5)
C2—N1	1.519 (3)	C14—H14	0.93
C2—C3	1.536 (4)	C15—C16	1.353 (5)
C2—H2A	0.97	C15—H15	0.93
C2—H2B	0.97	C16—C17	1.432 (5)
C3—C4	1.518 (4)	C17—C18	1.406 (5)
C3—C10	1.539 (5)	C17—C22	1.437 (4)
C3—H3	0.98	C18—C19	1.369 (5)
C4—C5	1.531 (5)	C18—H18	0.93
C4—C7	1.531 (4)	C19—C20	1.413 (6)
C4—H4	0.98	C19—H19	0.93
C5—C6	1.535 (4)	C20—C21	1.336 (7)
C5—H5A	0.97	C20—H20	0.93
C5—H5B	0.97	C21—C22	1.405 (6)
C6—N1	1.519 (3)	C21—H21	0.93
C6—H6A	0.97	C22—N13	1.364 (5)
C6—H6B	0.97	O12—H12	0.81 (4)
			
C2B—C1B—N1	115.8 (2)	C4—C7—H7B	109.9
C2B—C1B—H1BA	108.3	C8—C7—H7B	109.9
N1—C1B—H1BA	108.3	H7A—C7—H7B	108.3
C2B—C1B—H1BB	108.3	C7—C8—C9	113.3 (3)
N1—C1B—H1BB	108.3	C7—C8—N1	107.6 (2)
H1BA—C1B—H1BB	107.4	C9—C8—N1	114.1 (2)
C3B—C2B—C7B	116.8 (3)	C7—C8—H8	107.1
C3B—C2B—C1B	123.7 (3)	C9—C8—H8	107.1
C7B—C2B—C1B	119.3 (3)	N1—C8—H8	107.1
C2B—C3B—C4B	122.3 (3)	O12—C9—C16	110.3 (2)
C2B—C3B—Br2	121.2 (2)	O12—C9—C8	108.4 (2)
C4B—C3B—Br2	116.3 (2)	C16—C9—C8	110.5 (2)
C5B—C4B—C3B	119.2 (3)	O12—C9—H9	109.2
C5B—C4B—H4BA	120.4	C16—C9—H9	109.2
C3B—C4B—H4BA	120.4	C8—C9—H9	109.2
C4B—C5B—C6B	120.3 (3)	C11—C10—C3	128.9 (5)
C4B—C5B—H5BA	119.9	C11—C10—H10	115.6
C6B—C5B—H5BA	119.9	C3—C10—H10	115.6
C5B—C6B—C7B	120.0 (3)	C10—C11—H11A	120
C5B—C6B—H6BA	120	C10—C11—H11B	120
C7B—C6B—H6BA	120	H11A—C11—H11B	120
C6B—C7B—C2B	121.4 (3)	N13—C14—C15	124.8 (4)
C6B—C7B—H7BA	119.3	N13—C14—H14	117.6
C2B—C7B—H7BA	119.3	C15—C14—H14	117.6
N1—C2—C3	111.0 (2)	C16—C15—C14	119.4 (4)
N1—C2—H2A	109.4	C16—C15—H15	120.3
C3—C2—H2A	109.4	C14—C15—H15	120.3
N1—C2—H2B	109.4	C15—C16—C17	118.6 (3)
C3—C2—H2B	109.4	C15—C16—C9	119.4 (3)
H2A—C2—H2B	108	C17—C16—C9	122.0 (3)
C4—C3—C2	107.6 (2)	C18—C17—C16	125.3 (3)
C4—C3—C10	117.2 (3)	C18—C17—C22	117.4 (3)
C2—C3—C10	108.2 (3)	C16—C17—C22	117.3 (3)
C4—C3—H3	107.9	C19—C18—C17	121.5 (3)
C2—C3—H3	107.9	C19—C18—H18	119.2
C10—C3—H3	107.9	C17—C18—H18	119.2
C3—C4—C5	108.4 (3)	C18—C19—C20	119.8 (4)
C3—C4—C7	109.4 (3)	C18—C19—H19	120.1
C5—C4—C7	108.3 (3)	C20—C19—H19	120.1
C3—C4—H4	110.2	C21—C20—C19	120.5 (4)
C5—C4—H4	110.2	C21—C20—H20	119.7
C7—C4—H4	110.2	C19—C20—H20	119.7
C4—C5—C6	109.4 (2)	C20—C21—C22	121.4 (4)
C4—C5—H5A	109.8	C20—C21—H21	119.3
C6—C5—H5A	109.8	C22—C21—H21	119.3
C4—C5—H5B	109.8	N13—C22—C21	118.1 (3)
C6—C5—H5B	109.8	N13—C22—C17	122.7 (3)
H5A—C5—H5B	108.2	C21—C22—C17	119.2 (4)
N1—C6—C5	108.9 (2)	C2—N1—C6	107.4 (2)
N1—C6—H6A	109.9	C2—N1—C1B	111.48 (19)
C5—C6—H6A	109.9	C6—N1—C1B	110.6 (2)
N1—C6—H6B	109.9	C2—N1—C8	111.7 (2)
C5—C6—H6B	109.9	C6—N1—C8	105.9 (2)
H6A—C6—H6B	108.3	C1B—N1—C8	109.60 (19)
C4—C7—C8	109.1 (2)	C14—N13—C22	117.1 (3)
C4—C7—H7A	109.9	C9—O12—H12	108 (3)
C8—C7—H7A	109.9		
			
N1—C1B—C2B—C3B	94.4 (3)	O12—C9—C16—C17	−175.1 (3)
N1—C1B—C2B—C7B	−91.9 (3)	C8—C9—C16—C17	65.0 (3)
C7B—C2B—C3B—C4B	2.0 (5)	C15—C16—C17—C18	−178.6 (3)
C1B—C2B—C3B—C4B	175.8 (3)	C9—C16—C17—C18	2.3 (5)
C7B—C2B—C3B—Br2	−172.6 (2)	C15—C16—C17—C22	2.1 (4)
C1B—C2B—C3B—Br2	1.3 (4)	C9—C16—C17—C22	−177.0 (3)
C2B—C3B—C4B—C5B	−0.4 (5)	C16—C17—C18—C19	−175.3 (3)
Br2—C3B—C4B—C5B	174.4 (3)	C22—C17—C18—C19	4.1 (5)
C3B—C4B—C5B—C6B	−1.1 (6)	C17—C18—C19—C20	−1.9 (6)
C4B—C5B—C6B—C7B	0.9 (6)	C18—C19—C20—C21	−0.6 (7)
C5B—C6B—C7B—C2B	0.8 (6)	C19—C20—C21—C22	0.8 (7)
C3B—C2B—C7B—C6B	−2.2 (5)	C20—C21—C22—N13	179.5 (4)
C1B—C2B—C7B—C6B	−176.3 (3)	C20—C21—C22—C17	1.5 (6)
N1—C2—C3—C4	16.7 (3)	C18—C17—C22—N13	178.3 (3)
N1—C2—C3—C10	144.2 (3)	C16—C17—C22—N13	−2.3 (5)
C2—C3—C4—C5	−68.9 (3)	C18—C17—C22—C21	−3.8 (5)
C10—C3—C4—C5	169.1 (3)	C16—C17—C22—C21	175.6 (3)
C2—C3—C4—C7	49.0 (3)	C3—C2—N1—C6	49.6 (3)
C10—C3—C4—C7	−73.0 (3)	C3—C2—N1—C1B	170.9 (2)
C3—C4—C5—C6	49.8 (3)	C3—C2—N1—C8	−66.1 (3)
C7—C4—C5—C6	−68.8 (3)	C5—C6—N1—C2	−68.9 (3)
C4—C5—C6—N1	17.4 (3)	C5—C6—N1—C1B	169.2 (2)
C3—C4—C7—C8	−73.6 (3)	C5—C6—N1—C8	50.5 (3)
C5—C4—C7—C8	44.4 (3)	C2B—C1B—N1—C2	−64.3 (3)
C4—C7—C8—C9	150.5 (3)	C2B—C1B—N1—C6	55.2 (3)
C4—C7—C8—N1	23.3 (3)	C2B—C1B—N1—C8	171.6 (2)
C7—C8—C9—O12	−69.4 (3)	C7—C8—N1—C2	41.8 (3)
N1—C8—C9—O12	54.2 (3)	C9—C8—N1—C2	−84.9 (3)
C7—C8—C9—C16	51.5 (3)	C7—C8—N1—C6	−74.9 (3)
N1—C8—C9—C16	175.1 (2)	C9—C8—N1—C6	158.5 (2)
C4—C3—C10—C11	−29.2 (6)	C7—C8—N1—C1B	165.8 (2)
C2—C3—C10—C11	−150.9 (5)	C9—C8—N1—C1B	39.1 (3)
N13—C14—C15—C16	−2.8 (7)	C15—C14—N13—C22	2.6 (6)
C14—C15—C16—C17	0.2 (5)	C21—C22—N13—C14	−177.9 (4)
C14—C15—C16—C9	179.4 (3)	C17—C22—N13—C14	0.0 (6)
O12—C9—C16—C15	5.8 (4)	H12—O12—C9—C8	−149 (3)
C8—C9—C16—C15	−114.0 (3)		

### Hydrogen-bond geometry (Å, º)

**Table:**

*D*—H···*A*	*D*—H	H···*A*	*D*···*A*	*D*—H···*A*
O12—H12···Br1^i^	0.81 (4)	2.38 (4)	3.179 (2)	173 (3)
C2—H2*A*···Br2	0.97	2.91	3.406 (3)	113
C2—H2*B*···O12	0.97	2.32	2.997 (4)	126
C6—H6*A*···Br1	0.97	2.88	3.797 (3)	159
C7—H7*B*···O12	0.97	2.65	3.030 (4)	103
C15—H15···O12	0.93	2.32	2.697 (4)	104
C18—H18···Br1	0.93	2.96	3.758 (4)	145

Symmetry code: (i) *x*−1, *y*, *z*.
